# Primary Bone Marrow Hodgkin’s Lymphoma Debuting As Hemophagocytic Lymphohistiocytosis in a Patient With HIV

**DOI:** 10.7759/cureus.60864

**Published:** 2024-05-22

**Authors:** Anthony M Navarrete, Nicoletta Machin, Majd Jawad

**Affiliations:** 1 College of Medicine, Francisco Marroquín University, Guatemala City, GTM; 2 Hematology and Oncology, University of Pittsburgh Medical Center, Pittsburgh, USA; 3 Clinical Hematopathology, University of Pittsburgh Medical Center, Pittsburgh, USA

**Keywords:** classical hodgkin's, hemophagocytic lymphohistiocytosis (hlh), hiv aids, primary bone marrow lymphoma, lymphoma, hematology-oncology

## Abstract

The development of Hodgkin's lymphoma (HL) is a known complication in patients with human immunodeficiency virus (HIV) infection. Extranodal involvement, specifically primary bone marrow Hodgkin's lymphoma (PBMHL) is a rare manifestation that has been reported in HIV-positive patients and may represent a distinct entity from HIV-associated HL. We present a case of PBMHL presenting with hemophagocytic lymphohistiocytosis (HLH) in an HIV-positive patient. The 55-year-old male with HIV/AIDS presented with abdominal pain, diarrhea, and fever, leading to hospital admission. Despite initial treatment, he deteriorated, prompting re-admission and investigation revealing pancytopenia and elevated inflammatory markers, suggestive of HLH. Subsequent bone marrow biopsy unexpectedly revealed PBMHL. Treatment with HLH-directed therapy and the HLH-94 protocol resulted in significant clinical improvement. This case underscores the importance of recognizing atypical lymphoproliferative presentations in HIV/AIDS patients and the need for interdisciplinary collaboration in complex cases.

## Introduction

Primary bone marrow Hodgkin's lymphoma (PBMHL) presents a rare yet clinically significant manifestation, particularly observed in HIV-positive individuals, potentially delineating a distinctive subset within the spectrum of Hodgkin's lymphoma (HL) [1]. While HL is recognized as a prevalent malignancy (nearly 40% of all HIV patients) in the context of HIV infection, its classification as an acquired immunodeficiency syndrome (AIDS)-defining illness remains subject to ongoing debate and refinement [2]. The diagnostic journey of HIV-associated HL is often fraught with challenges owing to its infrequent presentation and symptom overlap with HIV-related complications, necessitating a nuanced approach to differential diagnosis [3]. Moreover, the intricate interplay between PBMHL and HIV introduces an additional layer of complexity, with the potential for the concurrent development of hemophagocytic lymphohistiocytosis (HLH), a hyperinflammatory syndrome, further complicating the clinical picture [4].

This report details a unique clinical case highlighting HLH as the initial manifestation of HL arising within the bone marrow in a patient with concomitant HIV infection. The intricate intertwining of these two entities underscores the necessity for a comprehensive understanding of their pathophysiological underpinnings and clinical implications, emphasizing the importance of vigilant surveillance and timely intervention in such complex scenarios.

## Case presentation

The patient is a 55-year-old male with a past medical history of HIV/AIDS since 2008 with poor adherence to antiretroviral therapy, paroxysmal atrial fibrillation, and esophageal candidiasis presented to the University of Pittsburgh Medical Center (UPMC) Presbyterian with two weeks of progressive abdominal pain, diarrhea, and fever. The patient presented to the emergency department as febrile and hypotensive. Intravenous fluids and ceftriaxone were administered. For personal reasons, he opted to leave against medical advice. He was discharged with levofloxacin 500mg PO for the treatment of his diarrhea. He presented to the emergency department two days later via ambulance with worsening non-bloody diarrhea and severe dehydration from an inability to tolerate oral intake. Laboratories at the time of admission demonstrated pancytopenia (WBC 3.3×10^9^/L, Hgb 8.5 g/dL, Plt 108 ×10^9^/L), serum ferritin 10,233 ng/dL, haptoglobin 301 mg/dL, lactate dehydrogenase (LDH) 380 IU/L, C-reactive protein (CRP) 25.8 mg/dL, triglycerides 179 mg/dL, D-Dimer 1.77 ug/mL and fibrinogen 564 mg/dL (Table 1). He was admitted to the medicine ward for further management. Hematology was consulted due to suspicion of HLH due to laboratory alterations. His HScore on admission was 203, which pointed to an 88-93% probability of HLH; in the setting of untreated HIV, there was concern the cytopenias were chronic in nature and not related to his acute presentation. The patient's hemodynamic status continued to decline, his laboratory derangement worsened, and he was transferred to the medical intensive care unit on day 2 of admission.

**Table 1:** Labs at the moment of admission WBC: white blood cells; Hgb: hemoglobin; Plt: platelets; LDH: lactate dehydrogenase; CRP: C-reactive protein

	Patient Value	Reference Range	Alert
WBC	3.3×10^9^/L	4.5 to 11.0 ×10^9^/L	
Hgb	8.5g d/L	12.9-16.9g d/L	
Plt	108 ×10^9^/L	150-370 x 10^9^/L	**
Ferritin	10,233 ng/dL	25-335 ng/dL	**
Haptoglobin	301 mg/dL	40-165 mg/dL	**
LDH	38 UI/L	140-280 UI/L	**
CRP	25.8 mg/dL	<0.3 mg/dL	**
Triglycerides	179 mg/dL	<150 mg/dL	**
D-Dimer	1.77 ug/dL	<0.50 ug/mL	**
Fibrinogen	564 mg/dL	200-400 mg/dL	**

**Figure 1:**
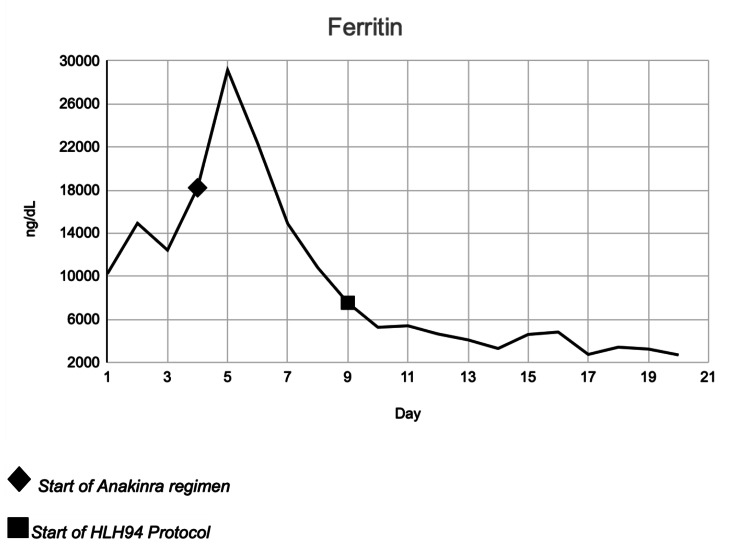
Ferritin levels throughout the course of admission

In the ICU, his clinical status continued to worsen, and his ferritin continued to rise; thus, a trial of anakinra 200 mg every eight hours IV and methylprednisolone 40mg IV were initiated on day four of admission. The patient's ferritin levels were at their peak at 28,000 ng/dL the following day. The trend in the patient's ferritin levels can be observed in (Figure 1). The trigger for HLH remained unclear (culture results/imaging), and peripheral blood smears were unremarkable. On day 6 of admission, all of his laboratory parameters started to demonstrate significant improvement, and the patient's hemodynamic and mental status began to return to his baseline, and a bone marrow biopsy was performed. On day 9 of admission, his clinical status again worsened after showing an initial downtrend in laboratory values between days 6-8. Due to this, the hematology team suggested the initiation of etoposide 74 mg/m^2^ and dexamethasone 10 mg/m^2^ per the HLH-94 Protocol. On day 10, after initiation of the HLH-94 protocol, the patient demonstrated remarkable clinical and significant laboratory improvement.

**Figure 2:**
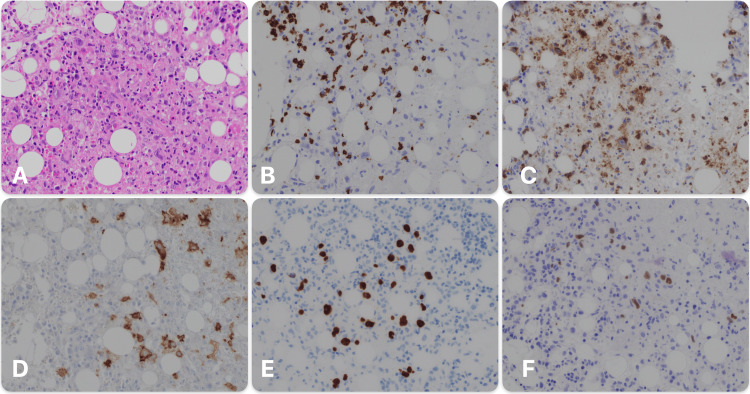
Bone marrow biopsy (A) Hematoxylin and eosin stain; (B) CD3 immunohistochemical stain; (C) CD15 immunohistochemical stain; (D) CD30 immunohistochemical stain; (E) PAX5 immunohistochemical stain

On day 12 of admission, the pathology department contacted the hematology team regarding the bone marrow findings. The bone marrow showed an atypical lymphoid infiltrate with large cells exhibiting Hodgkin/Reed-Sternberg-like features, including large lobulated nuclei with bi- and multinucleation and prominent nucleoli, immunohistochemistry and in situ hybridization were performed and the results were as follows: CD3: atypical cells were negative, CD15: atypical cells were partially positive, CD30: atypical cells were positive, Epstein-Barr virus in situ hybridization: atypical cells were positive, PAX5: atypical cells were weak to moderately positive (Figure 2). These findings, along with the absence of nodal involvement, led to a diagnosis of primary bone marrow HL.

Soon after the patient was transferred to the UPMC Hillman Cancer Center. He was scheduled to continue on his etoposide regimen and initiate AVD (doxorubicin, vincristine, and dacarbazine) therapy for his bone marrow HL; he received a dose of brentuximab vedotin (BV) as an outpatient. And is planned to receive a total of 12 cycles total with bone marrow biopsy and PET-CT after six cycles.

## Discussion

In our case, we described a patient with HIV-associated classic HL with primary bone marrow involvement complicated by HLH, leading to a delayed diagnosis and treatment initiation. The response to treatment for HLH is variable, as patients tend to respond only if the underlying trigger is effectively treated. The typical presentation of HL includes lymphadenopathy around the head, neck, axilla, or mediastinum, often accompanied by constitutional or "B-symptoms" such as drenching night sweats, fevers, and unexplained weight loss [1]. The patient mentioned in the case above did not present with these classic symptoms, and his underlying HIV/AIDS infection along with HLH development made the diagnosis complicated. In this patient, treatment was successful per the HLH-94 protocol, and his PBMHL is currently being treated, with the patient having completed his initial cycle of AVD without adverse effects. After his discharge from an inpatient setting, he received a dose of BV as an outpatient. As stated in other case reports and per the literature review, a combination of BV with AVD is superior to ABVD (adriamycin-bleomycin-vinblastine-dacarbazine regimen) or CHOP (cyclophosphamide, hydroxydaunorubicin, oncovin, prednisone) regimens. The use of BV+AVD demonstrated a 4.9 percentage-point lower combined risk of progression, death, or noncomplete response and use of subsequent anticancer therapy at two years [4].

HIV-associated HL typically presents with widespread disease involving unusual extranodal sites and follows an aggressive course. Isolated bone marrow involvement is uncommon and is more common in non-HL. PBMHL is rare and mostly reported in HIV-positive patients [3]. The patient's presentation was complicated by an initial presentation of HLH, which preceded the diagnosis of PBMHL. HLH is a rare syndrome characterized by uncontrolled immune system activation and a cytokine storm. The patient presented with an H score suggestive of HLH during his admission, highlighting the diagnostic challenge of overlapping symptoms in patients with HIV/AIDS.

Our literature review yielded six publications addressing PBMHL, reporting 11 additional patients; eight of those 11 patients were from the Ponzoni [2] study done in 2002. This retrospective study by Ponzoni et al. reported a median survival time in patients with HIV-associated PBMHL of four months compared with 15 months for patients with HIV-associated HL. It should be noted that the duration of onset of symptoms to the diagnosis of HIV-associated PBMHL lymphoma was one to five months in the literature. These features suggest that HIV-associated PBMHL is a clinically distinct entity with a more aggressive clinical course than HIV-associated HL [3]. Due to the rarity of this entity, most cases have been treated with the standard of care combination of BV+AVD and have shown adequate clinical response [5].

This patient's presentation was complicated by debuting in the hospital setting with a presentation of HLH before the diagnosis of PBMHL. HLH is a rare syndrome caused by an uncontrolled activation of the immune system and a cytokine storm. The patient presented with an HScore suggestive of HLH during his admission. However, due to his underlying HIV/AIDS, a diagnosis can be difficult because of overlapping symptoms. Further complicating the diagnoses is ferritin, being an inflammatory marker, can be elevated in both infection and HLH and has also been shown to be markedly elevated in patients with untreated/undiagnosed classic HL [4]. Our literature review found only two additional cases reporting PBMHL with HLH as a complication [6,7]. In those two cases and in our patient, HL in the bone marrow, along with the development of HLH, made this case challenging to manage and diagnose since the symptoms may overlap with each other. The clinical presentation in this case was consistent with HLH, although the criteria for diagnosing HLH can be debated. This contributed to initiating treatment with the HLH-94 protocol, which involves dexamethasone and etoposide [8]. The HLH-94 protocol, derived from primary HLH, has shown five-year survival rates of up to 62% in collaborative studies, but early mortality and neurological sequelae can occur in as many as 20% of cases [9,10]. While central nervous system (CNS) involvement is a concern in some types of lymphoma, it was not observed in our case. Both described cases underwent a similar course to ours, with the initiation of the HLH-94 protocol demonstrating a clinical improvement in the patient before starting his induction treatment for PBMHL.

## Conclusions

Patients with HIV and HL as a complication are often difficult to diagnose due to an atypical clinical course. The rare PBMHL that presents almost exclusively in HIV patients is even more difficult as the lack of nodal involvement makes the suspicion of HL low. Additionally, the combination of these pathologies is a trigger for HLH, which may further complicate the diagnosis and treatment of these cases.

## References

[1] Kaseb H, Babiker HM (2023). Hodgkin lymphoma. StatPearls [Internet].

[2] Ponzoni M, Fumagalli L, Rossi G (2002). Isolated bone marrow manifestation of HIV-associated Hodgkin lymphoma. Mod Pathol.

[3] Shah BK, Subramaniam S, Peace D, Garcia C (2010). HIV-associated primary bone marrow Hodgkin's lymphoma: a distinct entity?. J Clin Oncol.

[4] Connors JM, Jurczak W, Straus DJ (2018). Brentuximab vedotin with chemotherapy for stage III or IV Hodgkin’s lymphoma. N Engl J Med.

[5] Nagaharu K, Masuya M, Kageyama Y (2018). Successful treatment of primary bone marrow Hodgkin lymphoma with brentuximab vedotin: a case report and review of the literature. J Med Case Rep.

[6] Lin J, George B (2022). Primary bone marrow HIV-associated Hodgkin lymphoma complicated by hemophagocytic lymphohistiocytosis. Genes (Basel).

[7] Jordan MB, Allen CE, Weitzman S, Filipovich AH, McClain KL (2011). How I treat hemophagocytic lymphohistiocytosis. Blood.

[8] Bergsten E, Horne A, Aricó M (2017). Confirmed efficacy of etoposide and dexamethasone in HLH treatment: long-term results of the cooperative HLH-2004 study. Blood.

[9] Karakatsanis S, Panitsas F, Arapaki M (2022). Serum ferritin levels in previously untreated classical Hodgkin lymphoma: correlations and prognostic significance. Leuk Lymphoma.

[10] Lopez-Garcia A, Solan L, Alvarez B (2023). Hemophagocytic lymphohistiocytosis secondary to Hodgkin’s lymphoma with isolated bone marrow involvement in a newly diagnosed HIV patient. Medicina (Kaunas).

